# Effect of Hemp Hurd Biochar and Humic Acid on the Flame Retardant and Mechanical Properties of Ethylene Vinyl Acetate

**DOI:** 10.3390/polym15061411

**Published:** 2023-03-12

**Authors:** Mattia Di Maro, Maria Giulia Faga, Riccardo Pedraza, Giulio Malucelli, Mattia Bartoli, Giovanna Gomez d’Ayala, Donatella Duraccio

**Affiliations:** 1 Institute of Sciences and Technologies for Sustainable Energy and Mobility, National Council of Research, Strada delle Cacce 73, 10135 Torino, Italy; 2 Department of Applied Science and Technology, Politecnico di Torino, Viale Teresa Michel 5, 15121 Alessandria, Italy; 3 Consorzio Interuniversitario Nazionale per la Scienza e Tecnologia dei Materiali (INSTM), Via G. Giusti 9, 50121 Florence, Italy; 4 Center for Sustainable Future Technologies, Italian Institute of Technology, Via Livorno 60, 10144 Turin, Italy; 5 Institute for Polymers, Composites and Biomaterials, National Council of Research, Via Campi Flegrei 34, 80078 Pozzuoli, Italy

**Keywords:** EVA, hemp hurd biochar, flame retardance, humic acid, mechanical behavior

## Abstract

In this work, the combination of biochar produced through a pyrolytic process of hemp hurd with commercial humic acid as a potential biomass-based flame-retardant system for ethylene vinyl acetate copolymer is thoroughly investigated. To this aim, ethylene vinyl acetate composites containing hemp-derived biochar at two different concentrations (i.e., 20 and 40 wt.%) and 10 wt.% of humic acid were prepared. The presence of increasing biochar loadings in ethylene vinyl acetate accounted for an increasing thermal and thermo-oxidative stability of the copolymer; conversely, the acidic character of humic acid anticipated the degradation of the copolymer matrix, even in the presence of the biochar. Further, as assessed by forced-combustion tests, the incorporation of humic acid only in ethylene vinyl acetate slightly decreased both peaks of heat release rate (pkHRR) and total heat release (THR, by 16% and 5%, respectively), with no effect on the burning time. At variance, for the composites containing biochar, a strong decrease in pkHRR and THR values was observed, approaching −69 and −29%, respectively, in the presence of the highest filler loading, notwithstanding, for this latter, a significant increase in the burning time (by about 50 s). Finally, the presence of humic acid significantly lowered the Young’s modulus, unlike biochar, for which the stiffness remarkably increased from 57 MPa (unfilled ethylene vinyl acetate) to 155 Mpa (for the composite containing 40 wt.% of the filler).

## 1. Introduction

Polymer-based materials are known for their relatively high flammability [1]. The improvement of the fire behavior is a major challenge for extending advanced polymer applications, in particular in the fields where the flammability risk is high, such as the automotive, building, and electrical sectors.

Generally, most of the additives used as flame retardants for polymer-based materials comprise mineral, halogenated, inorganic nanometric fillers and/or phosphorus-, nitrogen-, and silicon-containing compounds; some of them may represent potential pollutants for the environment [2] and increase the chemical complexity of the final product, hence complicating its recycling [3]. For example, halogenated flame retardants can release highly toxic fire effluents into the atmosphere; for this reason, their use has been limited and, in some cases, forbidden [4,5]. Mineral flame retardants, such as metal hydroxides (especially of aluminum and magnesium), hydroxycarbonates, and zinc borates, need high loadings to achieve the desired flame-retardant properties and, therefore, can have a detrimental effect on the mechanical properties of the final product [2].

Due to the growing awareness of environmental issues associated with the increased use of fossil fuels [2,6], innovative flame retardant solutions based on renewable resources are currently generating a lot of interest from both the academic and industrial communities as follows: saccharide-based products (such as chitosan, lignin, cyclodextrin), bio-based aromatic compounds, deoxyribonucleic acids, proteins, phytic acid, and vegetable oils, have been tested as efficient green flame retardant additives thanks to their good char-forming ability [7,8,9,10,11,12,13].

Among the biomasses, humic acids (Has) are macromolecules that comprise organic matter resulting from the decay of vegetable and natural residues [14]. Commercial Has are extracted from peat and lignite and are widely available throughout the earth’s soil [14,15]. They consist of a skeleton of aliphatic and aromatic units stabilized in supramolecular structures by hydrogen bonds and weak hydrophobic interactions [16].

Humic acid has been tested as a flame-retardant additive in thermosetting polymers. In particular, Liu et al. [15] have studied the flame-retardant properties of HA chelated with different metal ions and incorporated into an epoxy resin. They found that the limiting oxygen index (LOI) increased from 21.2 (unfilled resin) to 26.6% when 10 wt.% of HA chelated with Fe was added. Venezia et al. [17] investigated the effect of HA (coupled with ammonium polyphosphate (APP) and urea) on the fire behavior of an epoxy resin and demonstrated that the presence of HA strongly improved the thermal stability of the composite by increasing charring. In addition, the formulation containing only 1 wt.% of phosphorus in the sample showed a quite surprising improved self-extinguishing capability.

Recently, biochar (BC), a solid carbonaceous residue recovered from the thermo-chemical conversion of waste biomass, has been explored as a possible flame-retardant additive. BC can be considered an inexpensive and viable alternative to traditional carbonaceous fillers for the development of polymer-based composites. In fact, biochar deriving from different waste biomasses has been used as a reinforcing filler for different polymers with the aim of improving their mechanical, thermal, and wear resistance properties [18,19,20,21,22,23]. Biochar can lead to the formation of a stabilizing protective char layer that acts as a barrier to the material’s combustion, making it a potential flame-retardant additive [24,25]. Moreover, the presence of inorganic salts in the biomass, which enrich the mineral composition of the biochar, can catalyze the depolymerization of the macromolecular chains, leading to the formation of stable condensed charred structures [2].

Pine wood (alone [21] and in combination with wool [24]), bamboo [26], oil seed rape [27], rice husk [27,28], and corn straw [28,29] derived biochars have been tested for improving polymer flame-retardant properties. In all the studied cases, a decrease in the peak heat release rate was observed because of the compact char layer that was effective in enhancing the insulation properties of the underlying polymer.

In this work, we thoroughly assess the effects of the concurrent presence of two biomass-based flame-retardant additives for ethylene vinyl acetate (EVA) copolymer, which is a material widely used in the automotive and electrical industry [30,31,32,33,34,35]. The flame-retardant additives are (i) biochar produced through pyrolysis of hemp hurd and (ii) commercially available humic acid (HA). The association of these two bio-based additives could lead to better resistance to irradiative heat flux with respect to that observed for the single component, as already proven for biochar and APP, employed as an intumescent flame retardant [24].

In a previous paper [19], we thoroughly investigated the mechanical, tribological, electrical, and thermal properties of EVA/fiber biochar composites at different filler loadings (from 5 to 40 wt.%). The incorporation of the fiber biochar into EVA copolymer accounted for a significant improvement of the thermo-oxidative stability of the polymer matrix, as well as for a higher thermal conductivity and microwave electrical conductivity with respect to the copolymer matrix, notwithstanding excellent wear properties (i.e., decreased friction coefficient and increased wear resistance). Conversely, the presence of the fiber biochar promoted a remarkable decrease in ductility when the filler loading exceeded 20 wt.%. Pursuing this research, in this work, we focused on the potential flame retardant behavior provided by biochar, trying, at the same time, to limit the worsening of the ductility induced by the filler itself. More specifically, we replaced hemp fiber biochar (i.e., which is derived from the plant stalks) with hemp hurd biochar (HHB, derived from the lignin-rich part of the hemp stems). The following two biochars differ also as far as the morphology is considered: hemp fiber biochar is made of short fibers (several millimeters long), while hemp hurd biochar is made of irregular micron-sized particles (granules). We prepared EVA composites containing HHB at two different concentrations (i.e., 20 and 40 wt.%) by melt mixing using an internal mixer. Further, we incorporated 10 wt.% of HA with respect to neat EVA and EVA/HHB composites, trying to assess the occurrence of joint or synergistic effects between the two additives. Their dispersion within the polymer matrix was investigated by SEM. The thermal, mechanical, and flame-retardant properties of the obtained composites were thoroughly evaluated. Therefore, this work aims for the following: (i) investigate the combination of hemp hurd-derived biochar and humic acid as key components of a flame retardant formulation for EVA copolymers and (ii) assess the effect of the concurrent presence of the two additives on the morphology, thermal and mechanical behavior of the obtained composites. In addition, to the best of the authors’ knowledge, humic acid has never been employed for flame retardant purposes in a thermoplastic polymer matrix as follows: the only examples regarding the use of HA in flame retardant formulations refer to epoxy systems [17].

## 2. Materials and Methods

### 2.1. Materials

Ethylene-vinyl acetate copolymer with 19% of vinyl acetate (VA) monomer (EVA Greenflex FF55, melt flow rate at 190 °C/2.16 kg of 0.7 g/10 min ISO 1133), supplied by Versalis. S.p.A (Mantova) was used as polymer matrix. Humic acid (sodium salt) was purchased by Alfa Aesar (in the form of about 200µm granules). The biochar (HHB), used as filler, was obtained by the pyrolysis of small hemp hurds provided by Assocanapa s.r.l. and was unsuitable for other applications.

### 2.2. Methods

#### 2.2.1. Hemp Hurd Waste Pyrolysis and Composite Preparation

Pyrolysis thermal treatment was performed in a tubular furnace (Carbolite TZF 12/65/550) at 1000 °C with a heating rate of 15 °C/min. The maximum temperature was retained for 30 min; then, it was cooled down to room temperature. All the pyrolysis treatment was carried out under N_2_ atmosphere with 0.4 mL/min flow rate. Composites were melt-compounded with a Brabender W50E (Plasti-Corder, Duisburg, Germany). Two HHB weight percentage loadings of HHB were selected, namely, 20 and 40 wt.% (composites hereinafter coded as EVA/HHB20 and EVA/HHB40). Besides, the amount of HA was set at 10 wt.% (composites hereinafter coded as EVA+HA, EVA/HHB20+HA, and EVA/HHB40+HA). Neat EVA was processed in the same way as the compounds containing the additives and employed as a reference. The melting chamber was firstly pre-heated at 120 °C, then the temperature was stabilized, and the raw materials (i.e., EVA, HA, and HHB) were simultaneously charged and mixed for 4 min at 70 rpm. At the end of the mixing process, the materials were cut into small pieces and then compression-molded with a Collin P200T press (Maitenbeth, Germany), working at 130 °C and 100 bar. Samples of different shapes were prepared for the following tests: 100 × 100 × 2.5 mm^3^ for cone calorimetry tests, and samples 1 mm thick suitable were prepared for tensile tests, thermogravimetric (TG), and differential scanning calorimetry (DSC) analyses.

#### 2.2.2. Fourier Transform Infrared (FT-IR) and Raman Analysis

Fourier transform infrared (FT-IR) analyses were carried out on neat and pyrolyzed hemp hurd using a Nicolet 5700 (Thermoscientific, Waltham, MA, USA) in attenuated total reflectance mode (ATR) using a Smartorbit (Thermoscientific, Waltham, USA) in the range from 550 to 4000 cm^−1^ with a resolution of 2 cm^−1^.

Raman spectroscopy tests were performed in the range from 500 to 3500 cm^−1^ using a Renishaw_Ramanscope InVia (H43662 model, Gloucestershire, UK) equipped with a green laser light source at 514 nm. The spectrum deconvolution of Raman spectra was made with a homemade software developed using Matlab^®^ (version R2020a) according to the procedure proposed by Tagliaferro et al. [36].

#### 2.2.3. Morphological Analysis (SEM and EDX)

To investigate the morphological features of pyrolyzed hemp hurd biochar and its compounds with EVA and study the distribution of the additives in the different combinations in the polymer matrix, scanning electron microscopy (SEM, ZEISS EVO 50 XVP—Oberkochen, Germany—with LaB_6_ source) was used. Samples were fractured in liquid nitrogen, and their cross-section was examined. In addition, charred residues after flame spread tests were investigated; their chemical composition was determined by EDX microanalysis (Oxford INCA Energy 200). To avoid charge effects during the analysis, a gold-metalized layer of ∼10 nm was applied to the samples.

#### 2.2.4. DSC and TGA

A differential scanning calorimeter (DSC, Q2000, TA instruments, New Castle, DE, USA, Mettler DSC822) was used to evaluate the thermal properties of all samples. Pure indium (ΔH_m_ = 28.15 J/g; T_m_ = 156.4 °C) was used as a standard for calibrating enthalpy and temperature. Crystallization temperature (T_c_), melting temperature ™, degree of crystallinity (%), and glass transition temperature (T_g_) were evaluated on about 8 mg of material for each sample. The experimental setup consisted of the following three cycles: from −40 °C to 150 °C (1st heating run), from 150 °C to −40 °C (cooling run), and lastly, from −40 °C to 150 °C (2nd heating run). The heating rate was 10 °C min^−1^ for all the ramps. The glass transition temperature was calculated as the maximum of the peak obtained from the first derivative curve. The crystallinity degree of EVA and its compounds was calculated using the ΔH method. Melting enthalpy of a 100% crystalline PE (ΔH_0_^PE^ = 297 J·g^−1^) [37] was used as standard, as crystallinity refers to polyethylene segments only.

Thermogravimetric analyses were carried out in the air and nitrogen atmospheres (gas flow: 45 mL/min) using a TGA Discovery (TA Instruments, New Castle, DE, USA). About 11 mg of samples were treated from 50 °C to 800 °C with a heating rate of 10 °C min^−1^. Thermogravimetric analyses revealed the temperature of the maximum rate of degradation (T_max_) measured as the peak of the first derivative of the TG curve and the temperature corresponding to 10% weight loss (T_10%_).

#### 2.2.5. Characterization of the Fire Behavior

The flammability of EVA and its composites was assessed through UL94 tests performed in vertical configuration.

Forced-combustion tests were carried out with a cone calorimeter from Noselab instrument (Nova Milanese, Italy), following the ISO 5660 standard. The samples (size 100 × 100 × 2 mm^3^) were exposed to a 35 kW/m^2^ irradiative heat flux in a horizontal configuration. Time to ignition (TTI, s), peak of heat release rate (pkHRR, kW·m^−2^), time to peak (s), total heat release (THR, kW·m^−2^), total smoke release (TSR, m^2^·m^−2^), specific extinction area (SEA, m^2^ kg^−1^), and the residues at the end of the tests were evaluated. At least three tests were performed for each type of material, and the results averaged. In order to study if HA exerted a synergic, additive or antagonistic effect with HHB in EVA matrix, the concept of synergistic effectiveness (E_S_), as proposed by Lewin and Weil [38] and their equation were used as follows:

(1) $$Es=\left\{{\left(F_{P}\right)}_{fr+s}-{\left(F_{P}\right)}_{p}\right\}/\left\{\left({\left(F_{P}\right)}_{fr}-{\left(F_{P}\right)}_{p}\right)+\left({\left(F_{P}\right)}_{s}-{\left(F_{P}\right)}_{p}\right)\right\}$$

where (F_P_)_p_ is the flame-retardant property of the polymer alone, (F_P_)_fr_ is that of the polymer containing flame retardant, (F_P_)_s_ is the flame-retardant property of the polymer containing synergist, and (F_P_)_fr+s_ is that of the polymer containing both the flame retardant and synergist. If E_S_ results >1, synergy occurs. For additive systems E_S_ = 1 for less than additive systems E_S_ < 1, and for antagonistic systems E_S_ < 0.

#### 2.2.6. Mechanical Properties

Tensile tests were performed with an Instron 5966 dynamometer (Norwood, MA, USA) on dumbbell specimens, following the ASTM D638 standard. Ten measurements were performed for each type of compound and the results were averaged. The distance between the clamps was set at 3 cm. For these experiments, the deformation speed for each specimen was the following: 1 mm/min until the 0.2% elongation (to measure Young’s modulus), then 50 mm/min until the breaking of the material. Tensile strength (MPa), Young’s modulus (*E*), and elongation at break (%) were recorded. The results obtained from these tests were subjected to Spearman correlation test and one-way ANOVA test (*p*-value < 0.05) using Excel™ 2020 software (Microsoft Corp., Redmond, WA, USA) and the “Data analysis” tool.

## 3. Results and Discussion

### 3.1. Morphological and Structural (FTIR and Raman) Analysis of Hemp Hurd-Derived Biochar

**Figure 1:**
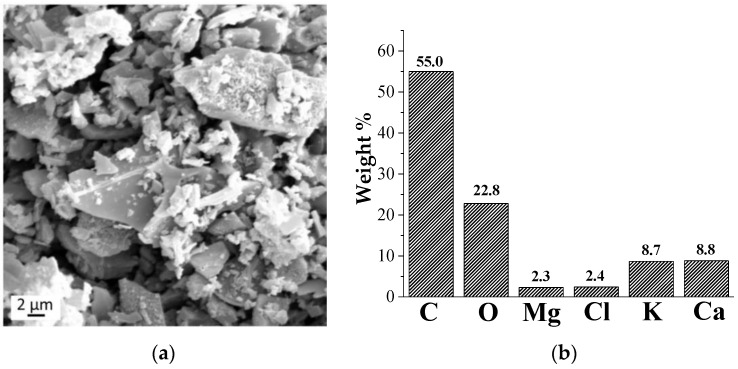
SEM micrograph (**a**) and EDX analysis (**b**) of the hemp hurd-derived biochar (HHB) at 1000 °C.

SEM observations and EDX micro-analysis were performed on hemp hurd biochar powder to evaluate the morphology and the chemical composition of the filler. Figure 1a shows a typical image of HHB filler where chips of irregular shape and dimension are visible. More in detail, the dimensions vary from the sub-micro- to the micro-scale ranging from half a micron to 40–50 µm. The elemental analysis (Figure 1b) indicates that, beyond carbon (C) and oxygen (O), calcium (Ca), magnesium (Mg), potassium (K), and chlorine (Cl) are identified. As expected, the amount of oxygen with respect to that of carbon is very low due to the high temperature used for the pyrolysis process (i.e., 1000 °C).

**Figure 2:**
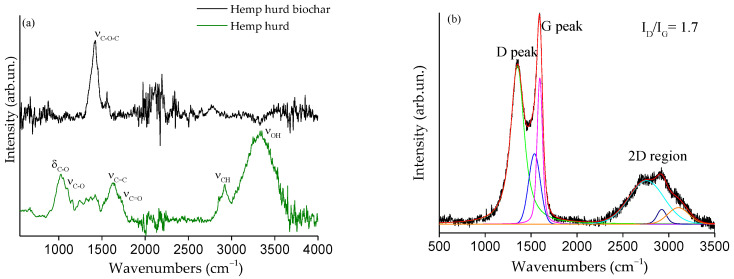
FT-IR spectra of (**a**) hemp hurd (green line) and hemp hurd biochar (black line) in the range from 500 to 4000 cm^−1^; (**b**) Raman spectrum of hemp hurd biochar in the range from 550 to 3500 cm^−1^.

The FT-IR spectrum (Figure 2a, green line) of hemp hurd shows a broad band between 3500 and 3000 cm^−1^ due to ν_OH_ of hydroxyl functionalities of cellulose and hemicellulose. The presence of lignin is also confirmed by the ν_C=C_ signal located at 1640 cm^−1^, while skeletal vibration of polysaccharides (ν_C-O_ and δ_C-O_) are observed at lower wavenumbers. Additionally, partial oxidation of the hemp hurd due to oxidative stress, air exposure, and simple aging is likely to be attributed to the presence of ν_C=O_ (carboxylic signals) at 1701 cm^−1^. Hemp-derived biochar shows a simpler spectrum (Figure 2a, black line), with only a band at 1418 cm^−1^ ascribed to the C-O-C mode of residual oxygen functionalities present on the biochar surface. The I_D_/I_G_ ratio of hemp hurd biochar is equal to 17, and its Raman spectrum (Figure 2b) shows a well-resolved D and G and bumped 2D region, suggesting a disordered graphitic-like carbon [39]. This is supported by the small size of the in-plane graphitic cluster diameter (L_a_, below 26 Å), calculated according to Tuinstra and Koenig [40].

### 3.2. Morphological Analysis of EVA+HA, EVA/HHB, and EBA/HHB+HA Composites

**Figure 3:**
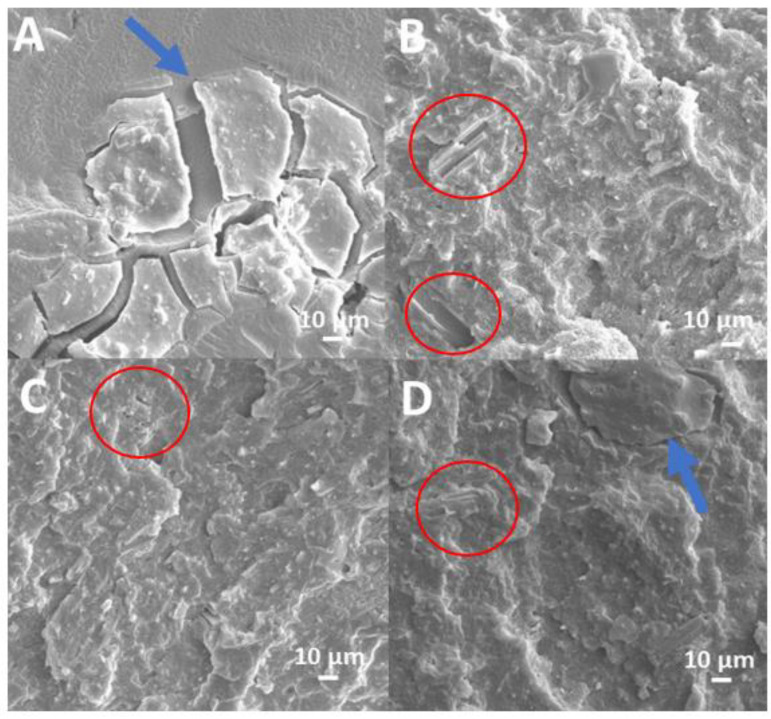
Typical SEM micrographs of fracture surfaces for EVA+HA (**A**), EVA/HHB 20 (**B**) and EVA/HHB40 (**C**), and EVA/HHB20+HA (**D**).

The SEM micrographs of cryogenically fractured surfaces for EVA composites are shown in Figure 3. Figure 3A presents the fracture surface of the EVA+HA composite where humic acid, which is in the form of sodium salt, the following is evident: it shows a structure consisting of flat sheets (indicated by blue arrows) not uniformly dispersed in the EVA matrix. These sheets also look detached and not well embedded in the polymer matrix because of the different polarities of the two phases. Conversely, in the composites containing HHB (Figure 3B,C for EVA/HHB20 and EVA/HHB40, respectively), the filler particles of sub-micro to the microscale are visible and uniformly distributed in the matrix (some of them are indicated with red circles). Further, they appear well embedded in the polymer matrix due to the graphitic-like carbon nature of biochar that is kindred to -C-C- polymer chains. As a matter of fact, both EVA and HHB show low polarity characteristics, also considering the low amount of VA units in the copolymer (i.e., 19%). In EVA/HHB40+HA, both HA flat sheets and HHB are visible (Figure 3D).

### 3.3. Thermal Analysis

**Figure 4:**
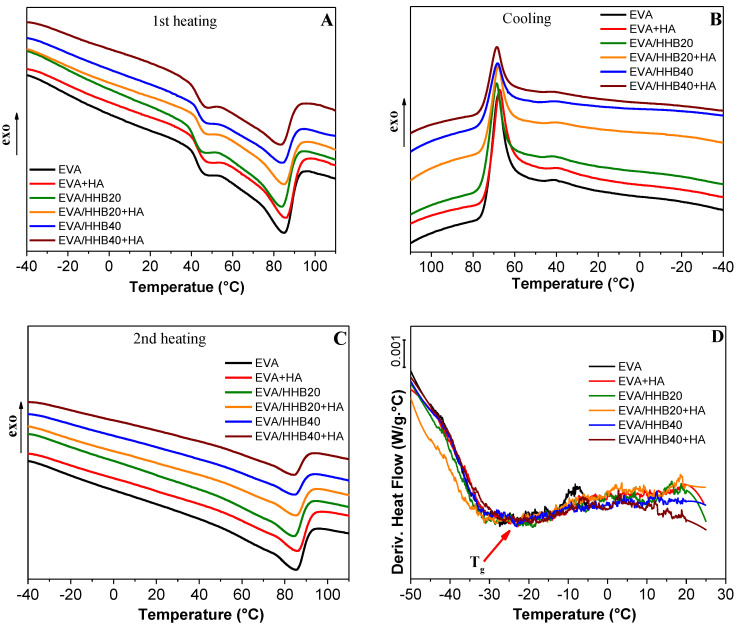
DSC curves of EVA and its composites (**A**) from −40 °C to 110 °C (**B**) from 110 °C to −40 °C and (**C**) from −40 °C to 110 °C (heat flow rate: 10 °C/min). (**D**) First derivative of the 1st heating run.

DSC thermograms of unfilled EVA and its composites are shown in Figure 4, whereas the thermal parameters are collected in Table 1. In the first heating run (Figure 4A), neat EVA shows two distinct melting peaks, at 46.5 °C and 85.4 °C, due to the presence of an amorphous fraction of VA that generates polyethylene chains with a different propensity to crystallize and, in turn, to melt [19]. After melting, the cooling run, reported in Figure 4B, shows the main crystallization of PE obtained mainly from the longer chains centered at about 68 °C and the second crystallization peak, visible at 41 °C, due to the crystallization of the shorter PE chains. Finally, the second heating run (Figure 4C) shows a single melting peak at 85.8 °C. The glass transition temperature (T_g_) of unfilled EVA, measured by the first derivative of the 1st heating run (Figure 4D), is at −25.4 °C. Overall, for all the composites, the presence of humic acid and/or HHB does not affect the thermal behavior of the polymer matrix, which demonstrates that neither humic acid nor biochar hinders the melting/crystallization process of unfilled EVA. At the same time, the T_g_ of the composites remains almost the same as the unfilled polymer (about −25 °C), confirming the poor interaction between the polymer matrix and the fillers.

The crystallinity degree (χ_c_) of the materials was also evaluated by DSC using, as a standard value, the ΔH_m_ of a 100% crystalline PE, taking into account the ΔH_m_ of composites measured in the first heating run. Unfilled EVA shows a crystallinity degree of 24%, a value comparable with the crystallinity degree found for other EVA copolymers with the same VA contents (i.e., 19% of VA monomer) [40].

As already noted in our previous work [19], the presence of biochar does not significantly influence the crystallinity degree of EVA (both composites containing 20 and 40 wt.% of HHB show a χc of 25%). The addition of humic acid to the matrix causes a slight decrease in the crystallinity of the polymer, regardless of the presence of biochar.

**Figure 5:**
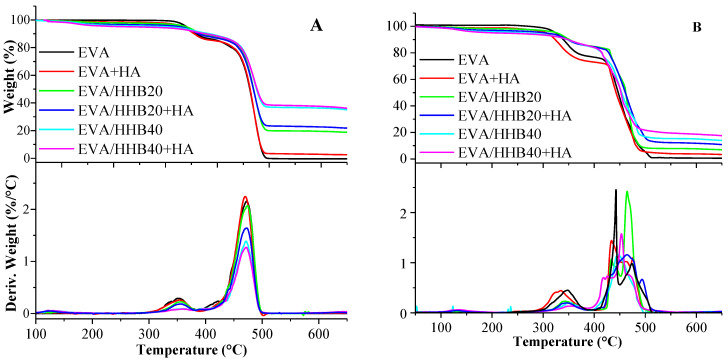
TG and dTG curves of EVA and its composites performed in (**A**) nitrogen and (**B**) air atmosphere. (heating rate: 10 °C/min).

The thermogravimetric curves of EVA and its composites, both in nitrogen and air, are shown in Figure 5A,B, respectively. The collected parameters are presented in Table 2. As widely reported in the literature [19,41], under a nitrogen atmosphere (Figure 5A), the degradation behavior of EVA copolymer is characterized by a two-step mechanism. The first step, with T_max1_ = 352 °C, involves the loss of the acetoxy groups related to the VA fraction. The resulting loss is about 15 wt.%, which is in agreement with the amount of the commercial EVA used in this work. The second step, characterized by a T_max2_ = 472 °C, is related to the degradation of the polyunsaturated chains derived by the first degradation step [42].

The addition of HHB increases the thermal stability of EVA; this increment refers to the HHB loading. This effect could be related to the low volatile nature of biochar that does not undergo further degradation processes in an inert atmosphere and could therefore act as a barrier to pyrolytic processes involving the polymer matrix. In fact, T_10_ is 359 °C for unfilled EVA and increases to 370 °C and 384 °C for EVA/HHB20 and EVA/HHB40, respectively. Less evident, albeit present, is the stabilizing effect of the filler on T_max1_ and T_max2_. In particular, T_max1_ increases from 352 °C (unfilled EVA) to 362 °C (EVA/HHB40 sample), while T_max2_ shows no substantial changes. Finally, the char residue (%) at 700 °C is in good agreement with the amount of HHB added to the polymer matrix.

The presence of humic acid (HA) does not seem to influence the thermal stability of the polymeric matrix. In fact, T_max1_ and T_max2_ remain practically unchanged, regardless of the presence of HA. Due to its chemical nature, which is more volatile than the polymer, the presence of HA promotes a decrease in T_10%_ values compared to the same samples without HA (i.e., from 359 °C of unfilled EVA to 355 °C for EVA+HA and from 384 °C for EVA/HHB40 to 376 °C for EVA/HHB40+HA).

Under oxidative conditions, the thermal behavior of EVA and its composites is different from that observed in nitrogen and is more complex. The incorporation of HHB into EVA leads to a general increase in the thermal stability of the matrix, as revealed by the significant increase in T_10%_ values (which shift from 339 °C for unfilled EVA to about 350 °C for the composites, irrespective of the HHB loading). Again, when HA is added to EVA, EVA/HHB20, or EVA/HHB40 composites, a decrease, often very small, in T_10%_ and T_max1_ is observed. The more complex oxidation of the C-C bonds that produce highly reactive radical species (such as hydroxyl and alkoxyl radical species). In fact, in the region between 400 and 550 °C, a multistep degradation process occurs. For this reason, T_50%_ is reported in Table 2 as an indication of the thermo-oxidative stability of these composites. Again, the presence of HHB enhances the stability of the composites at T_50%_, whereas no effect is visible in the presence of HA. These results agree with other findings [19,43].

### 3.4. Fire Behavior

The fire behavior of EVA and its composites was investigated by means of flammability (vertical flame spread) and forced combustion (i.e., cone calorimetry) tests. Vertical flame spread tests revealed a not classifiable rating for EVA and all its composites; indeed, the applied flame reached the sample clamp very quickly and was accompanied by an extended melt dripping.

**Figure 6:**
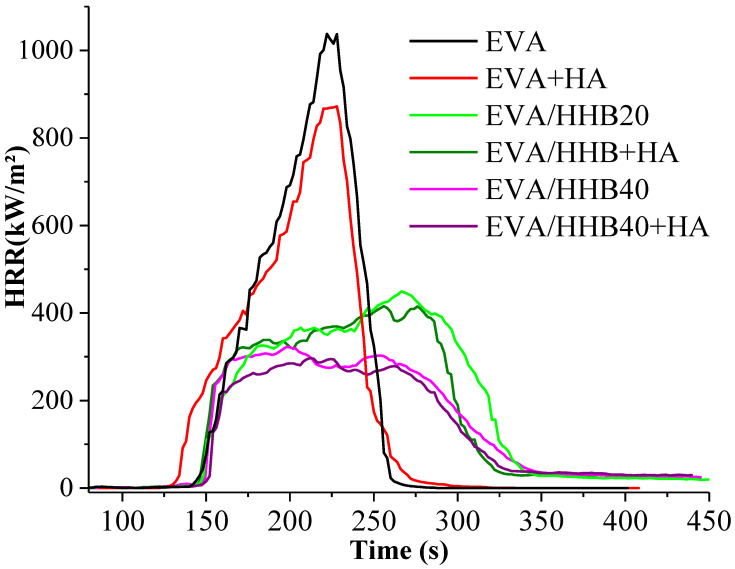
HRR vs. time cone calorimetry curves of EVA and its composites.

Table 3 reports the main thermal and smoke parameters collected during cone calorimetry tests carried out under 35 kW/m^2^ irradiative heat flux. Figure 6 shows the heating release rate (HRR) for EVA and all the composites. This thermal parameter is quite important because its reduction can be considered the most significant element in reducing fire loss.

Compared to unfilled EVA, the composites containing HA both with and without HHB show an anticipation of ignition (TTI), probably attributable to humic acid that, as many biobased flame retardants, starts activating before EVA degradation onset [12,13]. This finding is confirmed by the decrease in T_10%_ values in the presence of HA observed in thermogravimetric analyses, irrespective of the utilized atmosphere.

The presence of humic acid in EVA copolymer only slightly decreases both peaks of heat release rate and total heat release (by 16% and 5%, respectively), with no effect on the burning time. More significant is the effect due to the presence of HHB. The pkHRR and THR values are lowered to 69 and 29%, respectively, whereas the burning time increases by about 50 s for the composite with the highest HHB loading. These results can be ascribed to the protective effect exerted by the stable char originated during the forced-combustion test, as witnessed by the important increase in the residues at the end of the test. By adding HA to EVA composites, a further decrease in pkHRR and THR values can be observed. The pkHRR values have been chosen as the principal flame-retardant parameter (FP) to be used in Equation (1) for the calculation of the synergistic effectiveness, E_s_ [38,44]. We found that there is no synergistic effect between HA and HHB, but only a less than additive effect, i.e., E_s_ ~ 0.82 and 0.84 (Es < 1) for the EVA/HHB20+Ha and EVA/HHB40+HA composites, respectively.

**Figure 7:**
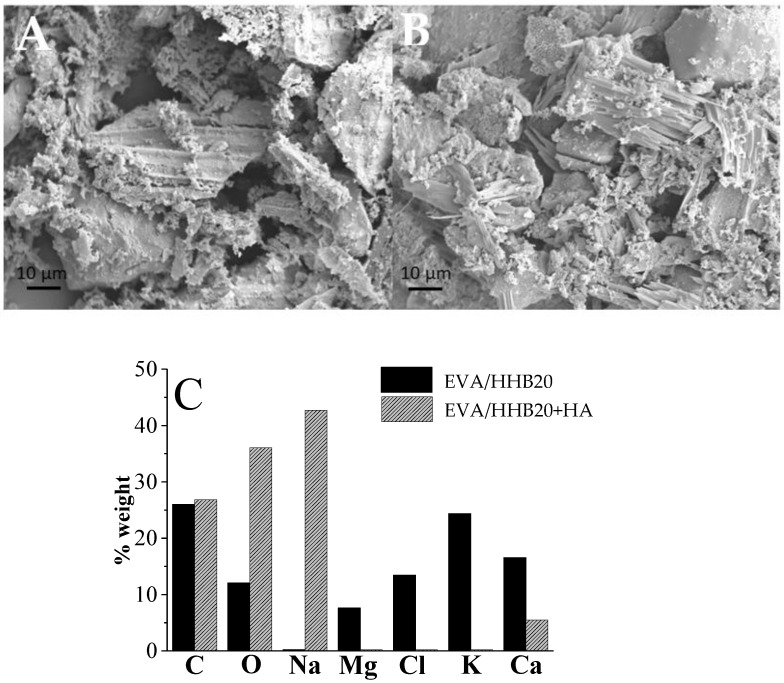
SEM micrographs of the residues after cone calorimetry tests for EVA/HHB20 (**A**), EVA/HHB20+HA (**B**), and their EDX analyses (**C**).

To observe the presence of a protective stable char originated during the forced-combustion test, residues are observed with SEM coupled with EDX. As an example, Figure 7A,B show these residues for EVA/HHB20 and EVA/HHB20+HA, respectively. In the composites containing HHB, the residues are mainly composed of biochar, as confirmed by elemental analysis (Figure 7C). Chips of different dimensions are visible and are characterized by the presence of bubbles on the surface due to the release of volatiles, which results in the expansion of the chips themselves. Similarly, in the EVA/HHB composites containing HA, the residues of HHB and HA appear in the form of small needles (Figure 7B).

### 3.5. Mechanical Properties

**Figure 8:**
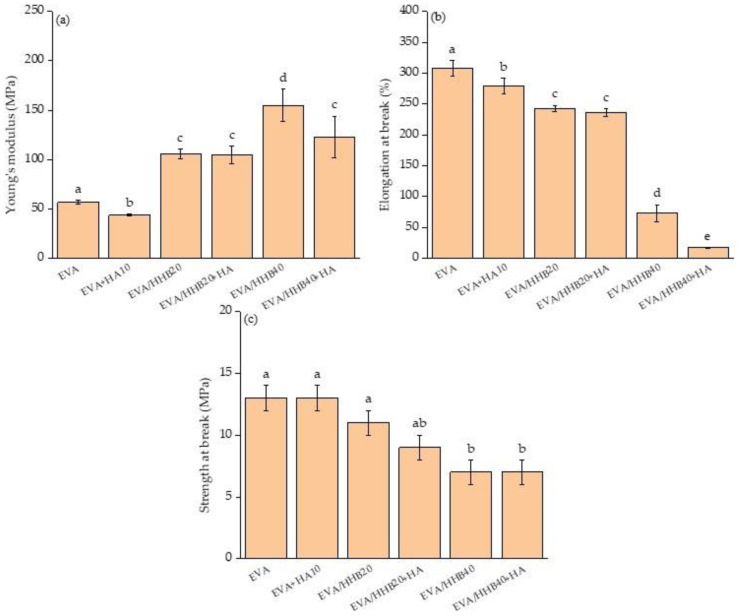
(**a**) Young’s modulus, (**b**) elongation at break, and (**c**) strength at break of unfilled EVA and its composites. Data annotated with different letters are significantly different (at 95% confidence level).

Young’s modulus, elongation, and strength at the break of unfilled EVA and its composites are reported in Figure 8a–c, respectively. The presence of HA significantly reduces Young’s modulus (E) and the elongation at break, whereas it does not impact the strength at break. On the contrary, in EVA/HHB composites, the Young’s modulus significantly increases as the filler content increases, from 57 MPa (unfilled EVA) to 155 MPa (EVA/HHB40 sample), whereas the strength at break (Figure 8c) decreases from 13 MPa (unfilled EVA) to 7 MPa (EVA/HHB40 sample). As expected, this stiffness increase accounts for a significant worsening in ductility, supporting a direct relationship between the HHB amount and the embrittlement of the composites. Besides, EVA/HHB40 shows a typical brittle fracture. This behavior was previously observed for both thermoset [18] and thermoplastic [19] matrices containing high loadings of biochar that acts as stress point, avoiding a redistribution of stress within the matrix. The addition of HA to EVA/HHB composites does not induce any further changes in the mechanical behavior of the composites. Only EVA/HHB40+HA sample shows a decrease in Young’s modulus with respect to EVA/HHB, being equal to the biochar loading.

## 4. Conclusions

In this work, we studied the effect of two biomass-derived flame retardant additives for ethylene vinyl acetate copolymer, namely, the following: (i) biochar produced through the pyrolytic process of hemp hurd and (ii) commercial humic acid. For this purpose, EVA/HHB composites at two different concentrations (i.e., 20 and 40 wt.%) and 10 wt.% of HA were prepared by melt mixing.

The presence of HA and/or HHB did not significantly impact the thermal behavior and the degree of crystallinity of the copolymer. Conversely, by increasing the HHB amount, the thermal and thermo-oxidative stability of EVA increased; at variance, the incorporation of HA in the copolymer accounted for anticipated degradation. The presence of HA in EVA only slightly decreased the peak of heat release rate and the total heat release (by 16% and 5%, respectively) without affecting the burning time. Further, the composites containing HHB showed decreased pkHRR and THR values (up to 69 and 29%, respectively), together with an increase in burning time of about 50 s when HHB was incorporated at the highest loading. Finally, the presence of humic acid significantly reduced the Young’s modulus, while HHB increased the composite stiffness, making its composite brittle.

A less than additive effect between the two bio-based flame retardant components was observed, where the main contribution was provided by HHB rather than HA. The proposed strategy seems interesting for the design of flame retarded systems based on bio-sourced flame retardant additives, better fulfilling the current circular economy concept.

## Figures and Tables

**Table 1:** Thermal parameters of EVA and its composites. (T_g_: glass transition temperature; T_m1_: melting temperature of the first heating run; T_c_: crystallization temperature; T_m2_: melting temperature of the second heating run; ΔH_m_: Enthalpy of fusion; χ_c:_ Crystallinity degree).

Samples	T_g_ (°C)	T_m1,1_ (°C)	T_m1,2_ (°C)	T_c1_ (°C)	T_c2_ (°C)	T_m2_ (°C)	ΔH_m1_ (J/g)	χ_c1_ (%)
EVA	−25.4	46.5	85.4	68.0	40.8	85.8	66.2	24
EVA+HA	−25.4	46.8	86.1	67.3	40.7	85.8	58.4	23
EVA/HHB20	−25.7	44.6	83.7	68.8	40.7	84.2	54.6	25
EVA/HHB20+HA	−25.8	46.0	84.7	68.1	40.6	85.3	42.8	21
EVA/HHB40	−25.4	46.7	83.9	68.4	41.2	83.7	40.9	25
EVA/HHB40+HA	−25.2	46.3	83.4	68.8	41.2	83.9	51.6	22

**Table 2:** Results from thermogravimetric analyses for EVA and its composites.

	N_2_				Air			
	T_10%_ (°C)	T_max1_ (°C)	T_max2_ (°C)	Residue (%) @ 700°C	T_10%_ (°C)	T_max1_ (°C)	T_50%_ (°C)	Residue (%) @ 700°C
EVA	359	352	472	0.1	339	350	442	0
EVA+HA	355	353	470	2.3	324	335	443	2.8
EVA/HHB20	370	358	475	18.5	352	343	462	6.9
EVA/HHB20+HA	364	358	473	21.4	347	342	461	9.8
EVA/HHB40	384	362	472	34.4	350	354	453	13.5
EVA/HHB40+HA	376	362	473	35.4	348	352	453	16.2

**Table 3:** Main thermal and smoke parameters by cone calorimetry tests.

	TTI (s)	pkHRR (kW/m^2^)	THR (MJ/m^2^)	SEA (m^2^/kg)	TSR (m^2^/m^2^)	CO/CO_2_ Ratio	Residue (g)
EVA	59 ± 1	1037 ± 63	62 ± 1	483 ± 5	903 ± 17	0.13 ± 0.03	0
EVA+HA	40 ± 5	872 ± 62	59 ± 2	511 ± 5	902 ± 22	0.03 ± 0.02	1.88 ± 0.01
EVA/HHB20	62 ± 4	450 ± 12	62 ± 1	467 ± 2	877 ± 29	0.08 ± 0.01	2.13 ± 0.11
EVA/HHB20+HA	61 ± 4	415 ± 25	58 ± 2	531 ± 30	919 ± 25	0.06 ± 0.01	2.98 ± 0.03
EVA/HHB40	56 ± 4	322 ± 24	50 ± 3	506 ± 33	774 ± 90	0.06 ± 0.02	4.77 ± 0.02
EVA/HHB40+HA	54 ± 7	294 ± 9	44 ± 1	383 ± 67	590 ± 50	0.05 ± 0.01	5.75 ± 0.01

## Data Availability

Not applicable.
